# Administering Antibiotics for Less Than Four Weeks Increases the Risk of Relapse in Culture-Positive Septic Arthritis of Native Joints

**DOI:** 10.3390/jcm12216808

**Published:** 2023-10-27

**Authors:** Eun-Jeong Joo, Bomi Kim, Kyung Mok Sohn, Sungmin Kym, Jungok Kim

**Affiliations:** 1 Division of Infectious Diseases, Department of Medicine, Kangbuk Samsung Hospital, Sungkyunkwan University School of Medicine, Seoul 03181, Republic of Korea; iamjoo@hanmail.net (E.-J.J.); springi02@naver.com (B.K.); 2 Division of Infectious Diseases, Department of Internal Medicine, Chungnam National University School of Medicine, Chungnam National University Hospital, Daejeon 35015, Republic of Korea; medone@cnuh.co.kr (K.M.S.); smkimkor@cnuh.co.kr (S.K.); 3 Division of Infectious Diseases, Department of Internal Medicine, Chungnam National University Sejong Hospital, Sejong-si 30099, Republic of Korea

**Keywords:** arthritis, infectious, septic arthritis, native joint septic arthritis, bone and joint infections, antibiotic duration

## Abstract

(1) Objectives: This study investigated the optimal duration of antibiotic therapy and determined the risk factors associated with relapse in patients with culture-proven septic arthritis of native joints. (2) Methods: A retrospective review was conducted on patients aged ≥18 years diagnosed with native joint septic arthritis, with bacteria isolated from joints and/or blood. The exclusion criteria were prosthetic joint infections and cases with no identified microorganisms. The outcomes were assessed in the remission and relapse groups. (3) Results: Among 479 patients with native joint septic arthritis, 137 met the inclusion criteria, with a median follow-up duration of 2.7 years. The relapse rate was 9.5%, which mainly occurred within 30 days after antibiotic treatment completion. Compared with the remission group, the relapse group showed a significantly higher proportion of cases that received antibiotic therapy for ≤ 4 weeks (4.8% vs. 46.2%, *p* < 0.001), synovial fluid white blood cell (WBC) counts ≥150 × 10^3^/mm^3^ (25.3% vs. 60.0%, *p* = 0.030), acute kidney injury (19.2% vs. 50%, *p* = 0.024), and extended-spectrum beta-lactamases-producing *Enterobacteriaceae* (0.8 vs. 15.4%, *p* = 0.024). Independent risk factors for relapse were determined as antibiotic therapy duration of ≤ 4 weeks (odds ratio (OR), 25.47; 95% confidence interval (CI), 1.57–412.33; *p* = 0.023) and synovial fluid WBC counts ≥150 × 10^3^/mm^3^ (OR, 17.46; 95% CI, 1.74–175.62; *p* = 0.015). (4) Conclusions: Patients with native joint septic arthritis require vigilant monitoring for relapse, particularly when treated with antibiotic regimens administered for less than four weeks or when synovial aspirates exhibit elevated WBC counts at diagnosis.

## 1. Introduction

Septic arthritis is a medical condition characterized by life-threatening illnesses and severe complications that trigger rapid joint destruction and an irreversible loss of joint function [1,2]. Effective management of septic arthritis necessitates a comprehensive approach, focusing on timely diagnosis and appropriate antibiotic therapy, in conjunction with early drainage procedures. Under current guidelines, patients with native joint septic arthritis are recommended to undergo antibiotic treatment for a duration of four to six weeks [3,4,5]. This recommended duration comprises an initial period of intravenous antibiotic administration, followed by a switch to oral therapy when clinical improvement is evident, although the precise timing for this switch has been a matter of ongoing debate [6,7,8]. While a well-designed study has addressed the possibility of short-course antibiotic therapy in conjunction with early drainage for native joint septic arthritis, the current level of recommended evidence to support this issue is low, despite a general consensus on a 4–6 weeks duration of antibiotic therapy and the timing of switching to oral therapy [3,9].

Several factors contributed to this uncertainty. Variables such as the specific type of joint affected, the presence of prosthetic materials, drainage methods, and host factors such as comorbidities and immune status may all impact treatment response [10,11,12]. Moreover, the diverse spectrum of causative microorganisms, from *Staphylococcus aureus* to multidrug-resistant microorganisms (MDROs), necessitates careful consideration when determining the appropriate treatment duration [13,14,15]. Furthermore, the literature presents various measurement parameters for evaluating treatment outcomes, including mortality and treatment failure, particularly in cases of repeated debridement procedures in patients whose clinical conditions do not improve [16,17,18]. This diversity in outcomes complicates guidelines, making it challenging for clinicians to reach a consensus on the appropriate duration of antibiotic therapy.

This study elucidates the optimal duration of antibiotic therapy for patients with culture-proven native joint septic arthritis. A retrospective study of adults with septic arthritis was conducted to identify the risk factors associated with relapse, and the findings can help clinicians optimize treatment regimens to achieve better outcomes while minimizing the risk of disease recurrence.

## 2. Materials and Methods

This retrospective review examined patients diagnosed with septic arthritis at a tertiary care university hospital in South Korea between 2004 and 2020. Patients were screened if they had been diagnosed with native joint septic arthritis as outlined by Newman [19]. The inclusion criteria were as follows: individuals aged ≥18 years, microorganisms isolated from joint fluid and/or blood, antibiotic therapy received for at least two weeks, and follow-up visits at least one month after completing the antibiotic treatment. The exclusion criteria were as follows: absence of identified microorganisms, isolation of *Mycobacterium* species, presence of joint prosthetic materials, suspected or confirmed osteomyelitis (e.g., diabetes mellitus foot ulcer), and lack of post-treatment follow-up. Cases involving death during treatment for septic arthritis were also excluded to prevent bias due to shortened antibiotic treatment duration.

Septic arthritis was defined as at least one of the following: isolation of a pathogenic organism from the affected joint, isolation of a pathogenic organism from another source (e.g., blood) along with a clinically swollen joint, typical clinical features consistent with septic arthritis and turbid joint fluid during ongoing antibiotic treatment, and suspicious pathological features indicative of septic arthritis [2,19]. The outcomes were categorized as remission (cure) and relapse. Relapse was defined as readmission and retreatment due to reaggravation in the previously affected joints after completing antibiotic therapy. This definition includes not only reinfection, characterized by a second infection by the same microorganisms but also persistent infection, indicated by the absence of isolated organisms and the presence of turbid fluid aspirated from the joint.

Data regarding demographics, comorbidities, clinical presentations, laboratory results at admission, causative microorganisms, medical and surgical management, and patient outcomes were collected. Drainage methods included repeated arthrocentesis and surgical debridement with arthroscopy or arthrotomy. The number of surgical debridement procedures was documented in cases where patients underwent surgery specifically due to persistent infection while concurrently receiving antibiotic therapy. Cases related to arthroplasty, arthrodesis, or amputation to improve joint function were excluded. Two clinicians meticulously reviewed medical records. An additional author cross-checked any discrepancies or conflicting data.

For the identification of microorganisms, both blood and joint fluid samples were promptly transported directly to the Microbiology Department of Laboratory Medicine at our hospital. The joint fluid samples, collected in sterile tubes with a minimum volume of at least 1 mL as per the laboratory’s requirements, were then immediately plated on agar plates, specifically blood agar plates, phenyl ethanol agar plates, and MacConkey agar plates. These plates were then incubated in incubators for up to 48 h at 37 °C. If colony growth was observed on the plates, they were sent to the microbiology laboratory for gram staining and identification. For blood samples, the BacT/Alert 3D automated blood culture analyzer (bioMérieux, Marcy L’Etoile, France) was consistently used throughout this study. Samples that generated a positive signal on the analyzer were expeditiously sent to the microbiology laboratory for gram staining and identification. Microbiological identification was performed using a standard identification card, and antimicrobial susceptibility testing was conducted using the modified broth microdilution method on a VITEK 2 automated system (bioMérieux, Marcy L’Etoile, France). We adhered to minimum inhibitory concentration breakpoints and quality-control protocols in line with the standards established by the Clinical and Laboratory Standard Institute (CLSI) [20]. The Laboratory Medicine Department at Chungnam National University Hospital maintains accreditation and undergoes annual inspections and surveys conducted by the Korean Society of Laboratory Medicine and the Korean Association of Quality Assurance for Clinical Laboratories.

Outcome groups were compared using the Pearson χ^2^ or Fisher’s exact test for categorical variables, and the Mann–Whitney test for continuous variables. A multivariate analysis of the risk factors for relapse was conducted using logistic regression analysis. Variables demonstrating medical or biological significance (*p* < 0.05) were included in the multivariate analysis. A receiver operating characteristic (ROC) curve analysis was used to assess the model’s validity. Data were analyzed using SPSS version 26.0 (IBM Corp. Armonk, NY, USA), with statistical significance set at *p* < 0.05 (two-tailed) deemed statistically significant.

## 3. Results

A total of 185 patients diagnosed with culture-positive septic arthritis in native joints were identified during the study period. The all-cause mortality rate in patients with septic arthritis confirmed with culture was 5.4% (10 of 185), and 38 patients were lost to follow-up. In total, 137 patients with septic bacterial arthritis were included in this study. The median follow-up period was 2.7 years (interquartile range, 0.6–6.7 years). The relapse rate was 9.5% (13 of 137); 12 patients experienced relapse within one month (range, 9–29 days) after completing antibiotic treatment, whereas a single case surfaced after 1.7 years, with *S. aureus* isolated. Among the thirteen relapse cases that led to readmission due to reaggravation of previously affected joints after discontinuing antibiotics, eight cases were identified with the same causative microorganisms and subsequently treated with antibiotics. In the remaining five cases, no microorganisms were isolated from the joint fluid, but pus-like fluid was evident upon aspiration. Four of these cases underwent surgical intervention after readmission, while one case was readmitted for retreatment without surgical intervention.

Table 1 provides details on the demographics, comorbidities, and management strategies. The median patient age was 64 years, with males accounting for 54% of cases. The comorbidities included diabetes mellitus (27%), osteoarthritis (11.7%), immunocompromised status (11.7%), and rheumatoid arthritis (6.6%), and there was no difference in underlying disease, including immunocompromised status, between the relapse and remission groups. The knee joint was the most commonly affected (46.7%), followed by the shoulder (23.4%) and hip joints (10.9%). The relapse group had more cases of hip involvement, whereas the remission group had more cases of shoulder involvement, although there was no significant difference in joint involvement between the two groups. Upon admission, approximately 40% of patients presented with fever, and nearly half of those whose blood cultures were collected (47.1%) demonstrated concurrent bacteremia. Notably, the relapse group demonstrated significantly higher white blood cell counts (WBC) ≥150 × 10^3^/mm^3^ (60% vs. 25.3%, *p* = 0.030) in the synovial aspirate. Acute kidney injury was also more prevalent in the relapse group than in the remission group (50% vs. 19.2%, *p* = 0.024).

Microorganisms were identified in the joint fluid cultures of all patients, except for two who exclusively had a pathogen isolated from their blood. The majority of the causative microorganisms were Gram-positive bacteria (89.1%), predominantly *Staphylococcus aureus*, with methicillin-resistant *S. aureus* (MRSA) constituting 30% (27 of 90) of the cases. Among the 15 cases of Gram-negative bacilli (GNB), three (20%) were attributed to extended-spectrum beta-lactamase (ESBL)-producing *Enterobacteriaceae*, which are responsible for community-onset infections. Although the causative microorganisms did not differ significantly between the two groups, the relapse group had two cases of ESBL-producing *Enterobacteriaceae* (15.4% vs. 0.8%, *p* = 0.024). Twenty-three patients (24%) experienced delays in receiving appropriate antibiotic therapy.

Drainage was performed in 87.6% of the patients, with initial drainage performed within 24 h of diagnosis in 42.3% of these cases. Among the fourteen cases of repeated arthrocentesis, six (42.9%) subsequently required arthroscopic debridement, ranging from 5 to 28 days after diagnosis. Although the modes and timing of drainage were consistent between the two groups, the relapse group demonstrated a tendency toward a higher frequency of repeated surgical debridement. Additionally, 26 underwent surgical debridement more than twice, of whom 13 (9.4%, 13 of 137) required a secondary operation after 4 weeks while receiving antibiotic treatment. The total duration of antibiotics was similar between the two groups. Patients who received appropriate antibiotics within 48 h accounted for 75.9%, and there were no significant differences between the relapse and remission groups. Of the twelve cases (8.8%) who were administrated antibiotic therapy for ≤4 weeks, the relapse group comprised a significantly higher proportion of cases compared with the remission group (4.8% vs. 46.2%, *p* < 0.001). There was no significant difference in terms of joint involvement between the two groups.

**Figure 1:**
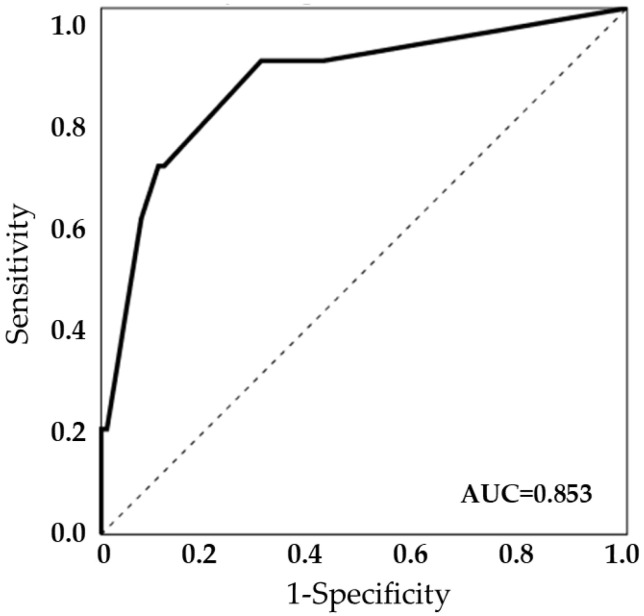
The model’s validity using a receiver operating characteristic (ROC) curve (area under the curve, AUC = 0.853).

Table 2 presents the variables that are significantly associated with relapse in patients with native joint septic arthritis. According to the univariate analysis, predisposing factors for a poor outcome included the total duration of antibiotic therapy for ≤4 weeks, synovial fluid WBCs, acute kidney injury, and ESBL-producing GNB. However, the initial drainage methods were not associated with relapse. In the multivariate analysis, a total duration of antibiotic therapy for ≤4 weeks (odds ratio (OR), 25.47; 95% confidence interval (CI), 1.57–412.33; *p* = 0.023) and synovial fluid WBCs ≥150 × 10^3^/mm^3^ (OR, 17.46; 95% CI, 1.74–175.62; *p* = 0.015) were identified as independent risk factors associated with relapse. The model’s validity was further assessed using an ROC curve, which yielded an area under the curve of 0.853, indicating the model’s predictive power (Figure 1).

## 4. Discussion

Managing septic arthritis is paramount in patient care and focuses mainly on optimizing the treatment duration to mitigate the risk of relapse. Our study aimed to address the knowledge gap regarding the uncertainty surrounding the optimal duration of antibiotic treatment for septic arthritis in native joints. Our findings indicate that a total antibiotic therapy duration of ≤4 weeks and a synovial fluid WBC count of ≥150 × 10^3^/mm^3^ may increase relapse risk.

Current guidelines recommend antibiotic treatment for at least 4–6 weeks for native joint septic arthritis with prompt drainage procedures [3,4,5,14]. Furthermore, this recommended duration encompasses intravenous administration over the initial 1–2 weeks, followed by a switch to oral therapy if viable and if the patient shows improvement [21]. Nevertheless, robust data needed to define the proper treatment duration according to specific pathogens or administration routes is scarce [6]. A recent randomized controlled trial proposed that a two-week course of antibiotic treatment following surgical drainage was as efficacious as a four-week regimen. However, this finding is particularly pertinent to hand and wrist joint arthritis [9]. It is plausible that septic arthritis affecting small joints could respond to a shorter duration of antimicrobial treatment, considering the comparatively more favorable outcomes compared with large joints [22]. Our study population comprised patients with a majority of large joints devoid of prosthetic materials. The antibiotic treatment of ≤4 weeks was correlated with relapse in culture-confirmed cases of bacterial septic arthritis in native joints, which suggests that careful vigilance is necessary in subgroups with a high risk of relapse after the completion of antibiotic treatment. Close follow-up and ongoing monitoring should be considered to promptly detect signs of relapse, and shorter treatment courses may be appropriate for specific groups.

This study identified an increased synovial fluid WBC count as an important predictor of relapsed septic arthritis. Previous research also indicated a connection between elevated synovial WBC counts and the need for repeated surgical interventions [23]. This elevation may reflect a high burden of infection and inflammation, particularly in large joints [24]. Such escalated levels may signify a more aggressive joint condition, resulting in persistent infection due to inadequate pathogen elimination, or relapse due to residual pathogens even after washout. Consequently, this can necessitate extended antibiotic therapy and additional interventions due to the insufficiency of a single debridement procedure [16]. Our observation reveals a strong correlation between elevated synovial fluid WBC counts and an increased risk of relapse in native joint septic arthritis. This suggests that synovial WBC counts may offer valuable information to clinicians for predicting outcomes not only during the treatment but also after its completion. Furthermore, these findings highlight the importance of a multidisciplinary approach to managing septic arthritis, involving close collaboration among specialists. Coordinated efforts can enhance the precision of treatment decisions and improve patient outcomes.

In our study, the distribution of pathogenic microorganisms causing septic arthritis is consistent with previous studies [12,13,22,25,26,27]. *S. aureus* was the most prominent organism, followed by *Streptococci* spp., and the proportion of Gram-negative bacteria was in concordance with the aforementioned studies. Overall, MRSA accounted for 20%, which aligns with epidemiologic data in Korea [25]. Although our hypothesis assumed a possible influence of causative microorganisms on relapse rates, we did not find any significant associations in this study. Virulent microorganisms or MDROs can exacerbate joint inflammation when patients experience delayed administration of appropriate antibiotics and lack of prompt drainage. This leads to poor outcomes and can result in joint damage or long-term sequelae. Studies have reported various relationships between microorganisms and distinct outcome measures, which are often influenced by differences in study design or heterogeneity in the studied population [26,28,29,30,31]. For instance, MRSA has been linked to increased mortality, but its role as a risk factor for treatment outcomes remains conflicting [32]. GNB, though infrequent pathogens, usually cause infections in elderly or immunocompromised patients, which could act as confounding variables, whether due to microbial factors or other underlying diseases [31]. Interestingly, we observed three cases (2.2%) of ESBL-producing GNB. Although this proportion is too small to have clinical implications, the emergence of multidrug-resistant pathogens raises concerns when selecting empirical treatment regimens based on community epidemiological data [2].

The microbiological diagnostic yield for septic arthritis has been a topic of interest, with certain studies reporting proportions as high as 80–90% [26,33]. However, the culture-positive rates may be lower when relying solely on joint specimen cultures [27,34,35]. Culture-negative results for synovial fluid may be altered by either the timing of synovial fluid aspiration and culture examination before the administration of antibiotic treatment or the time between synovial fluid aspiration and culture examination. Therefore, in an effort to detect causative microorganisms by obtaining joint fluid and/or blood samples before antibiotic administration, clinicians need to be cautious in cases of alternative diagnosis in culture-negative septic arthritis since it can sometimes unveil as other forms of acute arthritis, such as reactive arthritis, gout, or rheumatoid arthritis [4]. In our study, patients were initially screened by reviewing culture results of synovial fluid samples in the microbiology database. After the screening process, we enrolled patients based on the definitions of native joint septic arthritis following the inclusion and exclusion criteria. We conducted an exhaustive review of cases with positive culture results and included them in our study population. However, cases with negative culture results were not further evaluated to determine whether they represented native joint septic arthritis. As a result, we were unable to provide culture-positive rates for septic arthritis in native joints in the current study.

This study has certain limitations. First, its retrospective design introduces inherent biases and missing data, possibly affecting the accuracy of our findings. Second, the relatively small sample size may have reduced the ability to detect subtle associations. However, the extensive data collection period bolstered the robustness of the findings. Moreover, our focus was solely on culture-proven septic arthritis cases, excluding instances of inflammatory arthritis, which could have affected the applicability of our results to a broader population. Third, the absence of sequelae assessments, including functional outcomes, restricted our capacity to comprehensively measure treatment success, as our primary emphasis was bacterial eradication to prevent relapse. Finally, the group receiving antibiotic treatment for over 6 weeks constituted more than half of the study population. This diverse subgroup represents a common clinical scenario where repeated drainage was necessary due to persistent infection in specific patient groups.

In conclusion, our study provides evidence of the optimal duration of antibiotic therapy for treating septic arthritis in native joints. With an emphasis on an antibiotic treatment duration of less than four weeks and elevated synovial fluid WBC counts at diagnosis, physicians should be cautious in monitoring relapse risks when treating patients with native joint septic arthritis.

## Figures and Tables

**Table 1:** Clinical characteristics of patients with native joint septic arthritis.

	Remission (*n* = 124)	Relapse (*n* = 13)	Total (*n* = 137)	*p*-Value
Age, years	64 (54.3–73)	65 (54.5–73)	64 (54.5–73)	0.957
Male	67 (54.0)	7 (53.8)	74 (54.0)	0.990
Comorbidities				
Immunocompromised status	13 (10.5)	3 (23.1)	16 (11.7)	0.179 ^a^
Solid tumor	6 (4.8)	0 (0)	6 (4.4)	1.000 ^a^
Hematologic malignancy	0 (0)	1 (7.7)	1 (0.7)	0.095 ^a^
End-stage renal disease	4 (3.2)	0 (0)	4 (2.9)	1.000 ^a^
Liver cirrhosis	5 (4.0)	0 (0)	5 (3.6)	1.000 ^a^
Diabetes mellitus	32 (25.8)	5 (38.5)	37 (27.0)	0.337 ^a^
Rheumatoid arthritis	7 (5.6)	2 (15.4)	9 (6.6)	0.204 ^a^
Osteoarthritis	16 (12.9)	0 (0)	16 (11.7)	0.363 ^a^
Charlson’s comorbidity index	2 (1–4)	3 (2–4)	2 (1.5–4)	0.397
Previous intra-articular injection	47 (37.9)	5 (38.5)	52 (38.0)	1.000 ^a^
Previous arthroscopic procedure	5 (4.0)	0 (0)	5 (3.6)	0.529 ^a^
Recent blunt trauma	23 (18.5)	3 (23.1)	26 (19.0)	0.712 ^a^
Hospital-acquired infection	5 (4.0)	1 (7.7)	6 (4.4)	0.457 ^a^
Involved joints				
Knee	58 (46.8)	6 (46.2)	64 (46.7)	0.966
Shoulder	30 (24.2)	2 (15.4)	32 (23.4)	0.732 ^a^
Hip	12 (9.7)	3 (23.1)	15 (10.9)	0.155 ^a^
Elbow	8 (6.5)	1 (7.7)	9 (6.6)	1.000 ^a^
Wrist	6 (4.8)	1 (7.7)	7 (5.1)	0.511 ^a^
Ankle	5 (4.0)	0 (0)	5 (3.6)	1.000 ^a^
Small joints	11 (8.9)	0 (0)	11 (8.0)	0.599 ^a^
Polyarthropathy	6 (4.8)	0 (0)	6 (4.4)	1.000 ^a^
Combined periarticular abscess	11 (8.9)	3 (23.1)	14 (10.2)	0.131 ^a^
Fever on admission ≥38.3 °C ^b^	45 (37.8)	7 (63.6)	52 (40.0)	0.115 ^a^
Laboratory test at admission ^b^				
WBC (× 10^3^/μL)	11.0 (8.7–15.0)	13.8 (12.2–14.7)	11.5 (8.9–14.9)	0.141
CRP (mg/dL)	12.6 (6.1–21.8)	13.4 (7.9–17.8)	12.7 (6.4–24.5)	0.466
ESR (mm/h)	65 (39.3–99)	79 (62–112)	67 (41–100)	0.213
Acute kidney injury	23 (19.2)	6 (50.0)	29 (22.0)	0.024 ^a^
Synovial fluid WBCs (×10^3^/mm^3^) ^c^	11.3 (5.6–800.0)	55.1 (2.4–486.9)	91.5 (2.4–800.0)	0.573
<50	31 (32.6)	2 (20.0)	33 (31.4)	0.500 ^a^
50–100	25 (26.3)	1 (10.0)	226 (24.8)	0.445 ^a^
100–150	15 (15.8)	10 (1.0)	16 (15.2)	1.000 ^a^
≥150	24 (25.3)	6 (60.0)	30 (28.6)	0.030 ^a^
Concurrent bacteremia ^b^	45 (46.9)	4 (50.0)	49 (47.1)	1.000 ^a^
Causative microorganisms ^d^				
Gram-positive organism	111 (89.5)	11 (84.6)	122 (89.1)	0.636 ^a^
Staphylococcus aureus	81 (65.3)	9 (69.2)	90 (65.7)	1.000 ^a^
MRSA	24 (19.4)	3 (23.1)	27 (19.7)	0.720 ^a^
Streptococcus spp.	24 (19.4)	3 (23.1)	27 (19.7)	0.720 ^a^
Gram-negative organism	13 (10.5)	2 (15.4)	15 (10.9)	0.636 ^a^
ESBL-producing GNB	1 (0.8)	2 (15.4)	3 (2.2)	0.024 ^a^
Polymicrobial infections	4 (3.2)	1 (7.7)	5 (3.6)	0.397
Appropriate antibiotics ≤ 48 h	94 (75.8)	10 (76.9)	104 (75.9)	1.000 ^a^
Initial drainage ^e^	108 (87.1)	12 (92.3)	120 (87.6)	1.000 ^a^
Time to drainage (days)	1.0 (0–2)	2.0 (0–4)	1.0 (0–3)	0.093
Drainage ≤ 24 h	53 (42.7)	5 (38.5)	58 (42.3)	0.766
Modes of drainag^e^				
Repeated arthrocentesis	13 (10.5)	1 (7.7)	14 (10.2)	1.000 ^a^
Arthroscopic I&D	73 (58.9)	10 (76.9)	83 (60.6)	0.205
Arthrotomy ^f^	22 (17.7)	1 (7.7)	23 (16.8)	0.659 ^a^
Surgical debridement ≥ two times ^g^	22 (17.7)	4 (30.8)	26 (19.0)	0.270 ^a^
Second surgical debridement after 4 weeks while receiving antibiotic therapy	10 (8.1)	3 (23.1)	13 (9.5)	0.109 ^a^
Duration of antibiotic therapy				
Total antibiotics (days) ^h^	53 (40–78)	41 (24.5–88.5)	52 (38.5–78)	0.926
≤4 weeks ^i^	6 (4.8)	6 (46.2)	12 (8.8)	<0.001
4–6 weeks	30 (24.2)	1 (7.7)	31 (22.6)	0.297 ^a^
>6 weeks	88 (71.0)	6 (46.2)	94 (68.6)	0.067

Data are expressed as numbers (%) unless otherwise indicated. Continuous variables are expressed as median (interquartile range). WBC, white blood cell; CRP, C-reactive protein; ESR, erythrocyte sedimentation rate; MRSA, methicillin-resistant Staphylococcus aureus; ESBL-producing GNB, extended-spectrum beta-lactamase-producing Gram-negative bacteria; I&D, incision and debridement. ^a^ Fisher’s exact test. ^b^ Implies missing data. ^c^ Certain data were not collected because of pus-like debris that hindered the counting of WBCs and obtaining laboratory results in four cases (one relapse and three remission), or the limited amount of fluid aspirated from the small joints for comprehensive analysis beyond culture in ten cases. ^d^ Microorganisms were isolated from the joint fluid of all patients, except for two patients who had *S. aureus* bacteremia. ^e^ Of the seventeen cases without drainage, one was associated with relapse. ^f^ Nine of the eleven cases with small joint involvement underwent arthrotomy. ^g^ Surgical debridement included arthrotomy, arthroscopic incision, and drainage (I&D). ^h^ The shortest duration of antibiotic treatment was 18 days. ^i^ Of the twelve cases, the distribution of affected joints was as follows: five cases involved the knee joint (with three relapse cases), four cases involved the shoulder joint (with two relapse cases), two cases involved the wrist joint (with one case relapse), and one case affected a small joint (metatarsophalangeal joint).

**Table 2:** Risk factors for relapse in patients with native joint septic arthritis.

Variable	Univariate Analysis		Multivariate Analysis	
	OR (95% CI)	*p*-Value	OR (95% CI)	*p*-Value
Total duration of antibiotic therapy ≤ 4 weeks	16.86 (4.31–65.97)	<0.001	25.47 (1.57–412.33)	0.023
Synovial fluid WBCs ≥150 × 10^3^/mm^3^	4.43 (1.15–17.07)	0.030	17.46 (1.74–175.62)	0.015
Acute kidney injury	4.22 (1.25–14.28)	0.021		
ESBL-producing GNB	22.36 (1.88–266.63)	0.014		

Variables with *p* < 0.05 were included in the multivariate analysis. WBC, white blood cell; ESBL-producing GNB, extended-spectrum beta-lactamase-producing Gram-negative bacteria; OR, odds ratio; CI, confidence interval.

## Data Availability

Not applicable.
